# Evaluating the impact of organisational digital maturity on clinical outcomes in secondary care in England

**DOI:** 10.1038/s41746-019-0118-9

**Published:** 2019-05-16

**Authors:** Guy Martin, Jonathan Clarke, Felicity Liew, Sonal Arora, Dominic King, Paul Aylin, Ara Darzi

**Affiliations:** 1 Department of Surgery & Cancer, Imperial College London, London, UK; 2 School of Public Health, Imperial College London, London, UK; 3 DeepMind, London, UK

**Keywords:** Health policy, Outcomes research

## Abstract

All healthcare systems are increasingly reliant on health information technology to support the delivery of high-quality, efficient and safe care. Data on its effectiveness are however limited. We therefore sought to examine the impact of organisational digital maturity on clinical outcomes in secondary care within the English National Health Service. We conducted a retrospective analysis of routinely collected administrative data for 13,105,996 admissions across 136 hospitals in England from 2015 to 2016. Data from the 2016 NHS Clinical Digital Maturity Index were used to characterise organisational digital maturity. A multivariable regression model including 12 institutional covariates was utilised to examine the relationship between one measure of organisational digital maturity and five key clinical outcome measures. There was no significant relationship between organisational digital maturity and risk-adjusted 30-day mortality, 28-day readmission rates or complications of care. In multivariable analysis risk-adjusted long length of stay and harm-free care were significantly related to aspects of organisational digital maturity; digitally mature hospitals may not only deliver more harm-free care episodes but also may have a significantly increased risk of patients experiencing a long length of stay. Organisational digital maturity is to some extent related to selected clinical outcomes in secondary care in England. Digital maturity is, however, also strongly linked to other institutional factors that likely play a greater role in influencing clinical outcomes. There is a need to better understand how health IT impacts care delivery and supports other drivers of hospital quality.

## Introduction

Reducing variation in care quality, improving outcomes and lowering cost continue to be significant challenges for all healthcare systems. There is growing evidence that health information technology (IT) is a potential solution to these challenges, and hospitals increasingly rely upon electronic systems to support the delivery of high-quality, efficient and safe care.^1^ Electronic systems may have a number of potential benefits over traditional paper-based approaches and can help support patient safety, reduce adverse events, improve clinical outcomes, lessen error, strengthen the quality and availability of information, support better decision-making and communication and foster improvements in workflow and culture.^2–9^

The majority of digital technology evaluations focus on narrow operational or process led benefits of specific technologies, typically predate recent advances in health IT or are focused on single institutions; only a handful evaluate the impact of multi-functional commercially developed systems within complex organisations.^4,10–12^ In order to fully examine the impact of new digital technology, it is therefore important to understand its broader impact in real-world settings. The digital maturity of a healthcare organisation is the extent to which its health IT is an enabler of high-quality care through supporting improvements to service delivery and patient experience. Digital maturity is multi-faceted, and encompasses not only technology, resource and capability but also the digital literacy, ability and motivation of staff and patients to use new technologies.^11,13^ There is a relative paucity of evidence evaluating the impact of digital maturity on meaningful outcomes at an organisational or health system level. Despite this, there is a growing body of evidence to suggest that digitally mature hospitals with fully electronic health records and advanced order entry and clinical decision support systems have fewer complications, lower mortality rates and reduced costs of care.^5,9,14^

Much of the published literature looking at the impact of health IT on performance and outcomes is focused on the US healthcare system. The influence of centrally mandated incentives to improve the adoption and meaningful use of health IT such as the $30 billion HITECH Act,^15–17^ together with the significant additional expenditure on healthcare seen in the US compared with other developed countries (17.2% of GDP vs. 9%^18^), and significant variation in access, equity and outcomes,^19,20^ means that drawing meaningful comparisons with other healthcare systems is challenging. In addition, evaluations of digital technology to date have typically focused on specific products or functions in distinct settings. In practice, digital technology is used within complex sociotechnical systems that do not operate using discrete independent processes, meaning vital aspects of their impact may be missed.^21^ In this study, we therefore seek to further understand the impact of organisational digital maturity on clinical outcomes in secondary care in England.

## Results

### Population characteristics

Population characteristics and standard descriptive statistics for all outcome variables and covariates are presented in Table 1. There are significant differences in staffing, workload and infrastructure across each of the participating hospitals. The mean aggregate of the total CDMI score for each organisation was 797 (range 324–1253, SD 174). There was significant variation among the hospitals in all domains of CDMI with a mean infrastructure score of 68 (range 20–100, SD 16.4), mean capability score of 354 (range 115–671, SD 116) and mean readiness score of 376 (range 177–495, SD 67). Whilst there was significant variation in CDMI scores, as expected less variation was seen between the outcome variables of choice: mean SHMI 1.00 (range 0.67–1.17, SD 0.09, IQR 0.12), mean READ 99.54 (range 83.54–111.15, SD 5.58, IQR 7.60), mean LLOS 98.85 (range 72.23–119.89, SD 10.47, IQR 15.74), mean HFC 93.8% (range 87.78–98.76, SD 1.93, IQR 2.00) and mean COC 3.10% (range 1.54–5.80, SD 0.75, IQR 0.82).

**Table 1:** Characteristics and descriptive statistics for 136 non-specialist NHS Trusts in England

	Hospital characteristics (*n* = 136)			
	Mean	Range	SD	95% CI
2016 CDMI score				
Readiness (/500)	376	177–495	67	364–388
Capability (/800)	354	115–671	116	333–374
Infrastructure (/100)	68	20–100	16.4	65–71
Total (/1400)	797	324–1,253	174	766–828
Outcome variables (2015–2016)				
SHMI	1.00	0.67–1.17	0.09	0.99–1.02
HFC	93.81	87.78–98.76	1.93	93.47–94.15
LLOS	98.85	72.23–119.89	10.47	97.00–100.71
COC	3.10	1.54–5.80	0.75	1.94–5.33
READ	99.54	83.54–111.15	5.58	87.27–110.36
Covariates (2015–2016)				
A&E attendances	132,789	44,727–416,419	67,642	120,814–144,764
Inpatient admissions	113,246	30,494–255,895	48,990	104,573–121,918
Total WTE Staff (all groups)	5425	1850–14,209	2676	4951–5898
Total clinical staff	5278	1517–14,209	2636	2023–13,102
Clinical/non-clinical staff ratio	0.99	0.72–1.56	0.140	0.97–1.02
Dr/nurse staff ratio	0.40	0.140–0.56	0.074	0.391–0.42
Cons/Jnr Dr staff ratio	0.78	0.545–1.55	0.133	0.753–0.800
Manager/clinical staff ratio	0.41	0.013–0.092	0.014	0.016–0.0783
Total general beds	779	226–1835	325	721–836
Total ITU beds	29	5–108	24	25–34
General bed occupancy	0.89	0.69–0.99	0.05	0.88–0.90
ITU bed occupancy	0.82	0.50–1.00	0.10	0.81–0.84
Doctor/bed ratio	1.01	0.193–3.54	0.72	0.88–1.14
Nurse/bed ratio	2.47	0.47–8.16	1.60	2.18–2.75
Academic status	32/136 (23.53%)			

### Organisational digital maturity and outcomes

For each selected outcome variable, the results from the respective univariate and multivariable analyses are presented in Table 2.

**Table 2:** Association between organisational digital maturity and selected clinical outcomes for 136 hospitals in England

Digital maturity	Univariate analysis			Multivariable analysis		
	Coeff (B)^a^	95% CI	*P*	Coeff (B)^a^	95% CI	*P*
Summary hospital-level mortality index (SHMI)						
Total CDMI	−7.42^−5^	(−1.63^−4^–1.49^−5^)	0.102	3.50^−5^	(−5.71^−5^–1.27^−4^)	0.454
Readiness	−2.54^−5^	(−2.52^−4^–2.01^−4^)	0.824	1.62^−4^	(−1.16^–4^–4.39^−4^)	0.251
Capability	−1.51^−4^	(−2.83^−4^–1.97^–5^)	0.025	−6.86^−5^	(−2.32^−4^–9.48^−5^)	0.407
Infrastructure	−2.27^−4^	(−1.18^–3^–7.22^−4^)	0.637	4.11^−4^	(−2.32^−4^–9.48^−5^)	0.541
Harm-free care (HFC)						
Total CDMI	3.37^−3^	(1.48^−3^–5.26^−3^)	0.001	2.42^−3^	(2.03^−4^–4.63^−3^)	0.033
Readiness	7.66^−3^	(2.85^−3^–0.01)	0.003	5.55^−3^	(−1.16^−3^–0.01)	0.104
Capability	4.36^−3^	(1.51^−3^–7.22^−3^)	0.003	1.96^−3^	(−1.99^−3^–5.92^−3^)	0.329
Infrastructure	0.02	(1.30^−3^–0.04)	0.037	−6.90^−3^	(−0.04–0.03)	0.672
Long length of stay (LLOS)						
Total CDMI	0.02	(5.63^−3^–0.03)	0.002	0.01	(2.78^−3^–0.2)	0.014
Readiness	0.04	(0.01–0.06)	0.006	0.03	(5.14^−3^–0.06)	0.099
Capability	0.02	(5.23^−3^–0.04)	0.009	7.12^−3^	(−0.02–0.03)	0.449
Infrastructure	0.10	(−8.97^−3^–0.21)	0.072	9.88^−3^	(−0.14–0.16)	0.7898
Risk of readmission (READ)						
Total CDMI	2.49^−3^	(−3.13^−3^–8.11^−3^)	0.383	3.08^−3^	(−3.53^−3^–9.69^−3^)	0.358
Readiness	2.90^−3^	(−0.01–0.02)	0.686	−2.10^−3^	(−0.02–0.02)	0.836
Capability	3.88^−3^	(−4.50^−3^–0.01)	0.361	5.69^−3^	(−6.14^−3^–0.02)	0.343
Infrastructure	0.03	(−0.03–0.09)	0.295	2.09^−3^	(−0.09–0.10)	0.966
Complications of care (COC)						
Total CDMI	5.83^−4^	(−1.56^−4^–1.33^−3^)	0.121	−1.05^−4^	(−6.98^−4^–9.08^−4^)	0.796
Readiness	7.60^−4^	(−1.11^-3^–2.63^−3^)	0.423	−9.43^−4^	(−3.37^−3^–1.49^−3^)	0.444
Capability	8.95^−4^	(−2.07^−4^–1.10^−3^)	0.111	−1.53^−4^	(−1.58^−3^–1.28^−3^)	0.833
Infrastructure	6.73^−3^	(−1.06^−3^–0.01)	0.09	6.86^−3^	(−4.77^−3^–0.02)	0.245

^a^Non-standardised coefficient

### Relative risk of long length of stay (LLOS)

There was a significant association between organisational digital maturity and the relative risk of a long length of stay. At the univariate level, both the readiness and capability domains and aggregate total CDMI score were significantly related. This relationship remained when the other institutional covariates were accounted for in the multivariable model with total CDMI score (B = 0.01, *p* = 0.014, 95% CI 2.78^–3–0.02^) significantly related to LLOS (*R*^*2*^ = 0.2934).

### Harm-free patient care episodes (HFC)

At a univariate level, there was a clear underlying relationship between organisational digital maturity and the provision of harm-free care. HFC was significantly related to all aspects of the CDMI tool at the univariate level. Importantly, this relationship remained at a multivariable level when institutional covariates were accounted for with total CDMI score (B = 2.42–3, *p* = 0.033, 95% CI: 2.03^–4–4.63–3^) significantly related to HFC (*R*^*2*^ = 0.1499).

### Summary hospital-level mortality index (SHMI)

There was no clear relationship between organisational digital maturity and risk-adjusted 30-day mortality for hospitals in England. At a univariate level, only the capability domain of the CDMI tool; however, this relationship was not present in the multivariable model (*R*^*2*^ = 0.3207).

### Relative risk of readmission (READ)

There was no significant association between any aspect of organisational digital maturity and the relative risk of readmission and at either the univariate or multivariable level for hospitals in England.

### Patient episodes featuring complications of care (COC)

There was no significant association between any aspect of organisational digital maturity and patient episodes featuring complications of care and at either the univariate or multivariable level for hospitals in England.

## Discussion

Using nationally representative data from 136 hospitals in England from 2015 to 2016, we found that aspects of organisational digital maturity are significantly related to the relative risk of a long length of stay for patients receiving inpatient care and the provision of harm-free care. However, the relationships are not clear-cut, and whilst digital maturity inevitably does play a role in influencing clinical outcomes, other institutional variables that too are strongly linked to digital maturity also play a significant part and may have a far greater impact.

A strength of this study is the large, nationally representative data that have been used. It is a pragmatic examination of 13,105,996 inpatient admissions over a 12-month period across 136 acute care providers in England. This represents a larger and more representative cohort than previously evaluated in similar studies.^5,9^ Furthermore, this is the first study to examine impact of organisational digital maturity on clinical outcomes in the English healthcare system.

There remains widespread variation in organisational digital maturity across hospitals in England, despite the drive for a fully electronic NHS being more than a decade old.^22^ Determinants of clinical outcomes at an institutional level are numerous and complex, and encompass a wide range of inter-related factors with multi-directional cause and effect. For example, well-staffed,^23,24^ high-volume hospitals^25,26^ with manageable bed occupancy levels^27,28^ all have consistently better quality outcomes. In addition to considering other institutional factors that influence outcomes, it is also important to be cognisant of the high cost of new digital technology when evaluating its impact. The cost and quality repercussions of health IT investment have not been fully established, and the return on investment from expensive IT projects may take many years to come to fruition and be problematic to verify.^29^ The pursuit of better digital maturity has multiple drivers and many of it's perceived benefits are potentially achievable through alternative means. Therefore, determining the specific influence of digital maturity on clinical outcomes is a challenging endeavour. This study has nonetheless demonstrated the potential power and utility of administrative datasets to investigate crucial questions regarding the true impact of digital investment in secondary care.

This analysis has suggested that once other relevant organisational factors are considered, there appears to be no significant relationship between organisational digital maturity and risk-adjusted mortality, risk of readmission nor complications of care. This study did, however, demonstrate a significant relationship between LLOS and both the readiness domain and the total aggregate digital maturity score; a relationship that was remained significant, although less pronounced at the multivariable level. Digitally mature hospitals have a greater number of patients with a risk-adjusted long length of stay. Again, it is likely that in this instance, organisational digital maturity is confounded by other relevant institutional factors which are also likely associated with digital maturity. Large academic teaching hospitals are likely to treat patients with more problematic health needs and undertake more complex interventions. Although patients with common conditions who are treated at these major academic teaching hospitals have significantly lower mortality rates^30^ and lower rates of failure to rescue incidents,^26^ the presence of a high-risk complex patient cohort may act to negatively influence overall length of stay overall. These large academic hospitals, typically located in urban areas, are also more likely to be digitally mature and have better health IT than small, non-academic rural institutions that may lack the resource, knowledge and skill to embark on large, expensive and complex IT projects which can drive better performance and higher quality.^15,31,32^

This analysis has also demonstrated a relationship between HFC and organisational digital maturity; digitally mature hospitals deliver a greater number of harm-free care episodes to their patients. There is evidence that the use of electronic systems and other digital innovations can significantly improve compliance with prophylaxis^33^ and reduce the incidence of VTE events,^34^ reduce the duration of unneeded catheterisation^35^ and subsequent incidence of CAUTI,^36^ improve the prediction and identification of pressure ulcers^37,38^ and support better risk assessments and reductions in the incidence of inpatient falls.^39,40^ However, it is likely that this relationship may be confounded by more important determinants of safe care. It is plausible that hospitals with better resources buy better IT which in turn directly influences care, for example through better access to clinical decision support, improved surfacing of relevant clinical data and superior interoperability with other health and social care organisations. Alternatively, the digital maturity of an organisation may directly influence the quality and completeness of data capture which in turn affects outcome reporting; more mature organisations have higher quality data. However, digital maturity does also plausibly act as a proxy for the provision of safe care. Effective and well-led organisations are likely to have robust policies and procedures in place to reduce harmful events and deliver safe care, deploy more sophisticated approaches to quality improvement, and have better resources and a more mature and developed approach to health IT.

Given these findings, it is important that future works seeks to unpick the complex systems present in order to establish the true nature, direction and strength of the relationships between organisational characteristics, the use of digital technology and clinical outcomes. This evidence is currently lacking, but is crucial to support an evidence-based approach to digital technology in order to maximise its’ potential impact and provide value for money when making decisions about future investment.

This study has suggested that aspects of organisational digital maturity may play a role in influencing clinical outcomes for patients in hospitals in England; these results, however, must be qualified within the limitations of the study. Limitations of the study include the use of routinely collected data and the pragmatic approach to analysis; the key limitation being the inability to attribute direct causality and only infer association. Determinants of clinical outcomes at a hospital level are numerous and complex, and consider a wide range of inter-related variables. It is therefore inevitably challenging to establish causal inference and account for all confounders that may be present. Routinely collected administrative data were used to allow a detailed exploration of hospital performance and quality across a number of levels of care with a high degree of accuracy.^41,42^ Although a range of plausible covariates were selected to provide the best estimates for any underlying relationship between organisational digital maturity and clinical outcomes, we were unable to account for the full range of potential confounders. Although our outcome measures were risk-adjusted, our patient-level controls were limited. Quantifying organisational digital maturity is also challenging, and the CDMI Tool has some limitations. A number of varied approaches to evaluating organisational digital maturity have been proposed,^43–45^ but only a limited number use a comprehensive assessment framework,^46^ such as that used by the CDMI tool. The CDMI tool has an inherent risk of reporting bias, given it is self-reported. There is also a possibility that the CDMI tool fails to pick up the key aspects of digital maturity that have a significant impact on outcomes, whilst simultaneously measuring important aspects that have no influence, such as investment in security; the failure to find a persisting significant relationship between digital maturity and clinical outcomes may be due to gaps in the CDMI measure rather than the absence of a relationship. In addition, the benefits from health IT investment may only be seen at the higher end of the maturity spectrum, and so will only be achievable for a small number of hospitals studied limiting the overall findings. Finally, the return on investment from health IT may take many years to become apparent and so may not be detected in the time-limited data examined.

Using nationally representative data from 136 hospitals in England, this study has shown that routine administrative data have the potential to provide valuable information to inform an evidence-based approach to health IT investment in secondary care. A measure of organisational digital maturity—the NHS Clinical Digital Maturity Index—has been shown to be significantly associated with patients receiving harm-free care, but importantly is also significantly associated with an increased risk of a long length of stay. Digital maturity is however also strongly related to other institutional factors and measures of quality, and so ascertaining a direct causal relationship with clinical outcomes is challenging. There is no apparent relationship between digital maturity and complications of care, readmission rates nor overall risk-adjusted mortality. Whilst digital maturity has a significant role to play in the delivery of high-quality care, other institutional factors play a greater and more significant role in influencing clinical outcomes with digital maturity plausibly acting as a proxy for well-run, high-performing organisations delivering complex care. The effective use of health IT can undoubtedly lead to improvements in care quality, however, to maximise its potential we must develop a deeper understanding about how it impacts care delivery in holistic real-world settings and how it acts to support and enable other drivers of hospital quality.

## Methods

### Study population

The data were collected from all 136 non-specialist NHS Trusts in England that provide acute care over a 12-month period from January 2015 to January 2016. This 12-month period covered data for a total of 13,105,996 emergency and planned admissions. The data were collected from multiple sources covering the same 12-month period to ensure a temporal relationship between all included data. The data collected included Clinical Digital Maturity Index (CDMI) scores from 2016 for each organisation, five specified outcome variables of interest and a further 15 relevant variables pertaining to staffing, infrastructure and workload. The quality and completeness of the data were good with missing data accounting for <0.2% of all items included. The data is non-identifiable, and was collected from publicly available administrative datasets; therefore, no specific ethical approval or patient-level informed consent was required. The Dr Foster Unit at Imperial College London has approval from the UK Health Research Authority to hold and analyse the data for research purposes (Ref. 15/CAG/0005).

### The NHS Clinical Digital Maturity Index

There are multiple ways to assess digital maturity.^11^ The Clinical Digital Maturity Index (CDMI) is a mandatory national benchmarking tool for NHS organisations in England that provides an objective assessment of their organisational digital maturity. Through a self-assessment framework run by NHS Digital—the national provider of IT and data services for the NHS in England—comprising 133 individual questions, with organisations reporting their digital maturity across three main themes—readiness, capability and infrastructure—together with an overall aggregate maturity score.^47^ Readiness scores refer to an organisation’s ability to plan, deliver and optimise its digital systems (e.g., leadership, governance and strategic alignment), the Capability score is an assessment of the digital capabilities that are available to an organisation (e.g., electronic ordering or medicines management) and the Infrastructure score evaluates the extent to which essential infrastructure is in place to support the delivery of their required digital capabilities (e.g., Wifi provision or single sign-on). Overall maturity scores are awarded on a scale of 0–1400 and vary from 324 to 1253 across the organisations studied with a mean score of 797. The CDMI tool, despite not having been formally validated is an integral part of the digital agenda in the English NHS and is an evidence-based, broad and holistic measure that is unique in its universal assessment of an entire health systems digital maturity compared with other methods.^11^ The tool provides a robust, evidence-based and standardised assessment that allows for the direct comparisons between different organisations to be made. The aim of the tool is to allow organisations to benchmark their performance against their peers, and identify key areas for development and improvement. The tool is summarised in the online Supplementary Information.

### Outcomes measures

All outcome data used were based upon publicly available hospital episode statistics and routine administrative data obtained from NHS Digital^48^ and the Dr Foster Unit^49^ at Imperial College London, aggregated at the level of the hospital. All the data are collected, maintained and are publicly accessible through NHS Digital. The quality and completeness of the data were good, with <1% of data missing. As previously highlighted, the better use of health IT in secondary care has been linked to fewer complications and lower mortality rates.^5,9,14^ In addition, the adoption of widely used and well-understood outcome measures widens the generalisability and usefulness of any findings presented. Therefore, five clinical outcomes that may be plausibly associated with digital maturity and which are widely utilised in health services research were selected. Risk-adjustment is performed at a patient level and is derived from sets of logistic regression models that include the available case-mix factors based on complete national-level datasets; the national expectation of an event acting as the comparator^50^:

- Summary Hospital-Level Mortality Index (SHMI)—a risk-adjusted measure of deaths in hospital or within 30 days of discharge.^51,52^
- Relative risk of readmission (READ)—the number of emergency readmissions within 28-days of discharge from hospital.^53,54^
- Relative risk of long length of stay (LLOS)—the number of hospital episodes associated with a risk-adjusted length of stay exceeding the upper quartile for all patients nationally with that episode type.^55,56^
- Percentage of harm-free patient care episodes (HFC)—provision of harm-free care (the absence of pressure ulcers, falls, catheter associated urinary tract infections (CAUTI’s) and venous thromboembolism (VTE)) across each day of a hospital admission via the NHS Safety Thermometer.^57^
- Percentage of patient episodes featuring complications of care (COC)—a composite quality measure based on the occurrence of potentially preventable safety events as defined by the Agency for Healthcare Research and Quality (AHRQ) risk-adjusted quality indicators.^58,59^

### Hospital covariates

In total, 15 covariates pertaining to staffing, infrastructure and workload that were identified as potentially influencing outcomes were selected a priori in order to control for potential confounders of the relationship between digital maturity and the above outcome measures. There is a firm body of evidence that staffing levels—be that total staff numbers, variation in staffing levels, skills mix or access to senior clinicians—have a significant impact on clinical outcomes.^23,24,26,60–67^ Hospital characteristics such as bed numbers, academic or teaching hospital status and quality of non-digital infrastructure such as increased capital expenditure or access to better non-digital technology have also be shown to directly influence patient outcomes.^26,30,32,67–71^ Finally, the impact of workload, volume and bed occupancy across a range of settings has also been shown to impact outcomes.^25,27,28,64,68,69,72–76^ The data were collated from publicly available datasets published by NHS Digital.^48^ Academic hospital status was defined as membership of the Association of UK University Hospitals.^77^ The 15 covariates selected were: the total number of staff, total number of clinical staff, clinical/non-clinical staffing ratio, doctor/nurse staffing ratio, consultant/junior staffing ratio, manager/clinical staffing ratio, doctor/bed number ratio, nurse/bed number ratio, total number of adult inpatient beds, total number of adult critical care beds, academic hospital status, number of A&E attendances, number of inpatient admissions, mean inpatient bed occupancy and mean critical care bed occupancy; these are summarised in Table 1.

### Statistical analysis

All variables were assessed for normality with no further transformations deemed to be required. All outcome variables were treated as continuous variables. Standard descriptive statistics for all variables were applied.

Each of the panel of 15 hospital-specific variables was examined in univariate regression against each of the hospital-level outcome measures and all domains of CDMI. Univariate regression was also performed for each hospital-level outcome measure against each of the three hospital-level domains of CDMI and hospital-level aggregate CDMI score. A summary of these univariate associations is provided in the online Supplementary Table 1.

All variables were subsequently examined for multicollinearity using the variance inflation factor (VIF), and three variables were removed from the final multivariable model due to high collinearity; nurse/bed ratio, the total inpatient admissions and total staff numbers. Twelve remaining variables from the panel with a significant relationship to either the outcome variables or CDMI scores and low collinearity were included in the final multivariable models as plausible confounders for the relationship between CDMI score and clinical outcomes. Multivariable linear regression featuring the panel of retained hospital-specific covariates was then performed for each of the selected outcome measures. Statistical significance was set at *p* = < 0.05. All statistical analyses were performed in Stata V15 (StataCorp LLC, College Station, TX, USA).

## Supplementary information

Supplementary Information

## Data Availability

The full dataset and other relevant material used and analysed in this study are available from the corresponding author upon request.

## Supplementary information

**Supplementary information** accompanies the paper on the *npj Digital Medicine* website (10.1038/s41746-019-0118-9).

## Code availability

All statistical analyses were performed in Stata V15 (StataCorp LLC, College Station, TX, USA). The relevant code is available from the corresponding author upon request.
