# Supplementary material for: Evaluating the impact of organisational digital maturity on clinical outcomes in secondary care in England
Source: NPJ Digit Med. 2019 May 16;2:41. doi: 10.1038/s41746-019-0118-9 (PMC6550220; doi:10.1038/s41746-019-0118-9)
Supplement: Supplementary file 1 — Supplementary Information [file 41746_2019_118_MOESM1_ESM.pdf]

Supplementary Information 1 - summary of the NHS Clinical Digital Maturity Index Assessment Tool

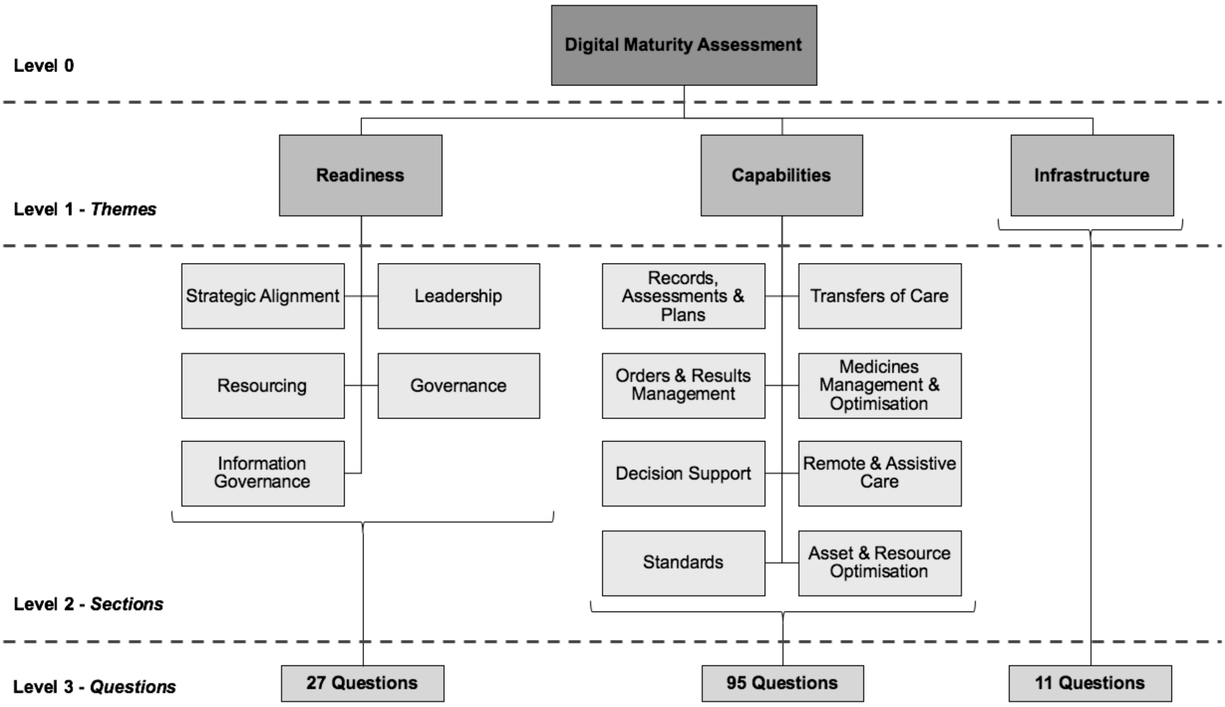

Supplementary Table 1 - univariate associations of 15 institutional covariates, 5 outcome variables and 4 CDMI variables

			Independent Variables				
			Outcome Variables (B, *p*, 95% CI)				
			SHMI	READ	LLOS	HFC	COC
Dependent Variables	CDMI Scores	Aggregate CDMI Score	-0.0000742, *p*=0.102 (-0.0001632 - 0.0000149)	0.0024879, *p*=0.383 (-0.0031329 - 0.0081087)	0.0155801, *p*=0.002 (0.0056274 - 0.0255328)	0.0033696, *p*=0.001 (0.0014758 - 0.0052635)	0.0005833, *p*=0.121 (-0.000156 - 0.0013227)
		Readiness Score	-0.0000254, *p*=0.824 (-0.0002516 - 0.0002007)	0.0029013, *p*=0.686 (-0.0112621 - 0.0170647)	0.0354604, *p*=0.006 (0.0102763 - 0.0606445)	0.0076534, *p*=0.00285 (0.0028515 - 0.0124552)	0.0007601, *p*=0.423 (-0.0011111 - 0.0026313)
		Capability Score	-0.0001513, *p*=0.025 (-0.0002829 - -0.0000197)	0.0038806, *p*=0.361 (-0.0044963 - 0.0122576)	0.0201933, *p*=0.0.009 (0.0052273 - 0.0351593)	0.0043619, *p*=0.003 (0.0015066 - 0.0072171)	0.0008948, *p*=0.111 (-0.0002068 - 0.0019964)
		Infrastructure Score	-0.000227, *p*=0.637 (-0.001176 - 0.00722)	0.0315014, *p*=0.295 (-0.0277588 - 0.0907617)	0.0984811, *p*=0.0.072 (-0.0089666 - 0.2059287)	0.0218572, *p*=0.037 (0.0012978 - 0.0424166)	0.0067342, *p*=0.090 (-0.0010569 - 0.0145252)
	Covariates	A&E Attendances	-3.09^{-7}, *p*=0.008 (-5.36^{-7} - -8.22^{-8})	-1.00^{-6}, *p*=0.892 (-0.0000156 - 0.0000136)	0.0000231, *p*=0.085 (-3.25^{-6} - 0.0000495)	6.36^{-6}, *p*=0.013 (1.35^{-6} - 0.0000114)	1.64^{-7}, *p*=0.867 (-1.77^{-6} - 2.10^{-6})
		Inpatient Admissions	-3.53^{-7}, *p*=0.027 (-6.64^{-7} - -4.10^{-8})	5.22^{-6}, *p*=0.604 (0.0000146 - 0.0000251)	0.0000354, *p*=0.053 (-4.35^{-7} - 0.0000712)	7.36^{-6}, *p*=0.036 (4.96^{-7} - 0.0000142)	9.36^{-7}, *p*=0.482 (-1.69^{-6} - 3.56^{-6})
		Total WTE Staff (all groups)	$-8.91^{-6,}$ *p*=0.002 (-0.0000146 - -3.27^{-6})	-0.000049, *p*=0.792 (0.0004148 - 0.0003168)	0.0013918, *p*=<0.0005 (0.0007667 - 0.002017)	0.000163, *p*=0.011 (0.0000376 - 0.0002885)	0.0000713, *p*=0.003 (0.0000244 - 0.0001182)
		Total Clinical Staff	-0.0000173, *p*=<0.0005 (-0.0000264 - -8.09^{-6})	-0.0001313, *p*=0.667 (-0.0007338 - 0.0004712)	0.0024027, *p*=<0.0005 (0.0013801 - 0.0034253)	0.0002974, *p*=0.005 (0.0000919 - 0.000503)	0.001178, *p*=0.003 (0.000406 - 0.000195)
		Dr/Nurse Staff Ratio	-0.334332, *p*=0.001 (-0.5376372 - -0.1310271)	-7.684432, *p*=0.250 (-20.84892 - 5.480057)	7.67461, *p*=0.538 (-5.066697 - 42.86107)	0.9752037, *p*=0.678 (-3.667653 - 5.61806)	2.295652, *p*=0.009 (0.5891436 - 4.00216)
		Clinical/Non-Clinical Staff Ratio	-0.2180829, *p*=<0.0005 (-0.3156949 - -0.1204708)	-1.37356, *p*=0.679 (-7.914914 - 5.167794)	5.91386, *p*=0.014 (3.010963 - 26.40413)	2.405923, *p*=0.037 (0.1444056 - 4.667441)	0.1121838, *p*=0.798 (-0.753939 - 0.9782966)
		Cons/Jnr Staff Ratio	0.0134597, *p*=0.817 (-0.1013511 - 0.1282706)	2.893469, *p*=0.427 (-4.283604 - 10.07054)	-20.3853, *p*=0.002 (-35.11527 - -9.971288)	-2.064643, *p*=0.105 (-4.566203 - 0.4369177)	0.214953, *p*=0.656 (-0.7364965 - 1.166403)
		Manager/Clinical Staff Ratio	-0.549033, *p*=0.31 (-1.614409 - 0.516343)	-34.7965, *p*=0.304 (-101.5338 - 31.94079)	7.109297, *p*=0.895 (-136.6396 - 108.233)	3.101841, *p*=0.795 (-20.42082 - 26.6245)	4.702077, *p*=0.294 (-4.129318 - 13.53347)
		Total General Beds	-0.0000444, *p*=0.066 (-0.0000916 - 2.91^{-6})	0.0012392, *p*=0.414 (-0.0017525 - 0.00422309)	0.0229891, *p*=0.001 (0.003491 - 0.140374)	0.0012596, *p*=0.017 (0.0002286 - 0.0022906)	0.0002884, *p*=0.15 (-0.0001054 - 0.0006823)
		Total ITU Beds	-0.0010909, *p*=<0.0005 (-0.0016822 - -0.0004995)	-0.150444, *p*=0.444 (-0.0538051 - 0.0237163)	0.1604666, *p*=<0.0005 (0.0949745 - 0.2259588)	0.013016, *p*=0.058 (-0.0004428 - 0.0264748)	0.0091411, *p*=<0.0005 (0.0042427 - 0.0140395)
		ITU Bed Occupancy	-0.1397301, *p*=0.079 (-0.2956875 - 0.162273)	-1.931775, *p*=0.700 (-11.81045 - 7.946899)	5.43295, *p*=0.552 (-12.60874 - 23.47464)	2.573182, *p*=0.142 (-0.8698438 - 6.016208)	1.515089, *p*=0.021 (0.2327551 - 2.797423)
		General Bed Occupancy	-0.2028078, *p*=0.18 -0.0948731 - 0.5004887)	-6.721321, *p*=0.479 (-25.46111 - 12.01847)	20.05866, *p*=0.198 (-8.305373 - 59.76003)	-1.552292, *p*=0.642 (-8.140063 - 5.035479)	1.369354, *p*=0.276 (-1.104528 - 3.843236)
		Dr/Bed Ratio	0.0321738, *p*=0.003 (0.0112487 - 0.0530989)	-0.6822582, *p*=0.319 (-2.032627 - 0.6681102)	-1.402548, *p*=0.263 (-3.868208 - 1.063113)	-0.6941698, *p*=0.003 (-1.155122 - -0.233218)	0.546871, *p*=0.547 (-0.2338336 - 0.1244593)
		Nurse/Bed Ratio	0.0151941, *p*=0.002 (0.0058554 - 0.245327)	-0.1709417, *p*=0.578 (0.7774672 - 0.4355837)	-2.299799, *p*=0.216 (-2.033345 - 0.1632269)	-0.2649029, *p*=0.013 (-0.4732815 - -0.0565242)	-0.0264339, *p*=0.516 (-0.1066754 - 0.0538076)
		Academic Status	-0.0602981, *p*=0.001 (-0.0949715 - -0.0256247)	-0.9780522, *p*=0.394 (-3.237948 - 1.281843)	9.578028, *p*=<0.0005 (5.773303 - 13.38275)	0.8289878, *p*=0.038 (0.0459312 - 1.1612044)	0.5918509, *p*=<0.0005 (0.309492 - 0.8742099)

			Independent Variables			
			CDMI Scores (B, *p*, 95% CI)			
			**Aggregate CDMI Score**	**Readiness Score**	**Capability score**	**Infrastructure Score**
**Dependent Variables**	**CDMI Scores**	**Aggregate CDMI Score**	-	-	-	-
		**Readiness Score**	-	-	-	-
		**Capability Score**	-	-	-	-
		**Infrastructure Score**	-	-	-	-
	**Covariates**	**A&E Attendance**	0.0005458, *p*=0.014 (0.0001133 - 0.0009782)	0.0001597, *p*=0.071 (-0.0000141 - 0.0003335)	0.0003794, *p*=0.011 (0.0000898 - 0.000669)	6.66^{-06}, *p*= 0.754 (-0.0000352 - 0.0000486)
		**Inpatient Admissions**	0.0009942, *p*=0.001 (0.000416 - 0.0015725)	0.0003277, *p*=0.006 (0.0000946 - 0.0005608)	0.0006089, *p*=0.002 (0.0002182 - 0.0009996)	0.0000576, *p*=0.045 (1.35^{-06} - 0.0001138)
		**Total WTE Staff (all groups)**	0.0222315, *p*=<0.0005 (0.0118101 - 0.32653)	0.0068479, *p*=0.002 (0.0025941 - 0.0111017)	0.0145655, *p*=<0.0005 (0.0075534 - 0.0215776)	0.0008181, *p*=0.123 (-0.0002233 - 0.0018595)
		**Total Clinical Staff**	0.03656, *p*=<0.0005 (0.193863 - 0.537338)	0.010551, *p*=0.004 (0.0035095 - 0.0175924)	0.0247527, *p*=<0.0005 (0.132468 - 0.362587)	0.0012563, *p*=0.15 (-0.0004615 - 0.0029741)
		**Dr/Nurse Staff Ratio**	267.1074, *p*=0.187 (-131.2956 - 665.5104)	21.18798, *p*=0.793 (-138.2692 - 180.6451)	220.5587, *p*=0.104 (-45.78657 - 486.904)	25.36068, *p*=0.186 (-12.37893 - 63.1003)
		**Clinical/Non-Clinical Staff Ratio**	156.0553, *p*=0.119 (-40.54736 - 352.6581)	-2.171525, *p*=0.793 (-81.08383 - 76.74078)	156.3026, *p*=0.019 (25.920332 - 286.6849)	1.924263, *p*=0.84 (-16.86754 - 20.71607)
		**Cons/Jnr Staff Ratio**	-0.4543243, *p*=0.997 (-218.514 - 217.6054)	15.72793, *p*=0.72 (-70.96212 - 102.418)	-29.64449, *p*=0.689 (-175.8321 - 116.5431)	13.46224, *p*=0.197 (-7.066112 - 33.99059)
		**Manager/Clinical Staff Ratio**	-295.2195, *p*=0.774 (-2325.478 - 1735.039)	48.33753, *p*=0.906 (-759.3901 - 856.0652)	-393.0792, *p*=0.569 (-1753.748 - 967.5897)	49.52212, *p*=0.611 (-142.6776 - 241.7218)
		**Total General Beds**	0.157739, *p*=<0.0005 (0.0709168 - 0.2445612)	0.0472616, *p*=0.009 (0.0120164 - 0.0825068)	0.0995344, *p*=0.001 (0.0409715 - 0.1580972)	0.010943, *p*=0.011 (0.0025366 - 0.193494)
		**Total ITU Beds**	2.251494, *p*=<0.0005 (1.138652 - 3.364336)	0.6809739, *p*=0.004 (0.2273325 - 1.134615)	1.470191, *p*=<0.0005 (0.7213576 - 2.219024)	0.100329, *p*=0.074 (-0.0098863 - 0.2105443)
		**ITU Bed Occupancy**	406.4855, *p*=0.007 (115.047 - 697.924)	62.475, *p*=0.3 (-56.20952 - 181.1595)	316.8003, *p*=0.002 (123.2541 - 510.3465)	27.21015, *p*=0.057 (-0.7875624 - 55.20787)
		**General Bed Occupancy**	-495.8152, *p*=0.084 (-1058.562 - 66.93161)	-258.277, *p*=0.023 (-480.2841 - -36.26988)	-190.6537, *p*=0.323 (-571.0073 - 189.6999)	-46.88445, *p*=0.084 (-100.1956 - 6.426748)
		**Dr/Bed Ratio**	-103.3575, *p*=<0.0005 (-140.4517 - -66.26341)	-38.06428, *p*=<0.0005 (-53.05485 - -23.07371)	-56.2904, *p*=<0.0005 (-82.1165 - -30.4643)	-9.002864, *p*=<0.0005 -12.5778 - -5.427932)
		**Nurse/Bed Ratio**	-44.24357, *p*=<0.0005 (-61.02527 - -27.46187)	-15.50511, *p*=<0.0005 (-22.3297 - -8.680521)	-24.8383, *p*=<0.0005 (-36.43264 - -13.24396)	-3.900166, *p*=<0.0005 (-5.511373 - -2.288958)
		**Academic Status**	123.9159, *p*=<0.0005 (58.57363 - 189.2581)	35.85577, *p*=0.009 (9.231871 - 62.47967)	80.46394, *p*=<0.0005 (36.48749 - 124.4404)	7.596154, *p*=0.02 (1.220212 - 13.9721)
